# Evaluating Trust Management Frameworks for Wireless Sensor Networks

**DOI:** 10.3390/s24092852

**Published:** 2024-04-30

**Authors:** Pranav Gangwani, Alexander Perez-Pons, Himanshu Upadhyay

**Affiliations:** Department of Electrical and Computer Engineering, Florida International University, Miami, FL 33174, USA; aperezpo@fiu.edu (A.P.-P.); upadhyay@fiu.edu (H.U.)

**Keywords:** internet of things (IoT), trust management, wireless sensor networks (WSNs), entropy, beta distribution

## Abstract

Wireless Sensor Networks (WSNs) are crucial in various fields including Health Care Monitoring, Battlefield Surveillance, and Smart Agriculture. However, WSNs are susceptible to malicious attacks due to the massive quantity of sensors within them. Hence, there is a demand for a trust evaluation framework within WSNs to function as a secure system, to identify and isolate malicious or faulty sensor nodes. This information can be leveraged by neighboring nodes, to prevent collaboration in tasks like data aggregation and forwarding. While numerous trust frameworks have been suggested in the literature to assess trust scores and examine the reliability of sensors through direct and indirect communications, implementing these trust evaluation criteria is challenging due to the intricate nature of the trust evaluation process and the limited availability of datasets. This research conducts a novel comparative analysis of three trust management models: “Lightweight Trust Management based on Bayesian and Entropy (LTMBE)”, “Beta-based Trust and Reputation Evaluation System (BTRES)”, and “Lightweight and Dependable Trust System (LDTS)”. To assess the practicality of these trust management models, we compare and examine their performance in multiple scenarios. Additionally, we assess and compare how well the trust management approaches perform in response to two significant cyber-attacks. Based on the experimental comparative analysis, it can be inferred that the LTMBE model is optimal for WSN applications emphasizing high energy efficiency, while the BTRES model is most suitable for WSN applications prioritizing critical security measures. The conducted empirical comparative analysis can act as a benchmark for upcoming research on trust evaluation frameworks for WSNs.

## 1. Introduction

Wireless Sensor Networks (WSNs) contain a multitude of sensors, gateways, microcontrollers, and routers [1]. These devices or sensors gather data from the physical surroundings and then wirelessly transmit them to an edge aggregator, a central hub, or gateway. WSNs are frequently used in applications such as smart transportation [2], industrial monitoring [3], wearable health monitoring for patients [4], military use, and many other domains where conventional wired communication is impractical.

WSNs contain sensors with constrained resources in terms of memory, power, and battery life [5]. These limitations make these sensors vulnerable to potential security threats [6] as they lack the capacity to run generalized security software [7]. Additionally, due to their reliance on wireless communication, WSNs are exposed to threats such as inception, interference, and disruption that may result in Denial-of-Service (DoS) attacks, data manipulation, and unauthorized access [8]. Furthermore, sensor nodes may also be vulnerable to destruction, physical attacks, and theft if the WSNs are installed in erratic and challenging surroundings. The establishment of trust becomes essential when a WSN consists of a multitude of sensors and edge aggregators that aggregate sensor data. This concept of trust is applicable not only within WSNs but also within the wider scope of the Internet of Things (IoT) [9]. This computation of trust offers various advantages such as the ability of the sink node (aggregator) to recognize malicious or malfunctioning sensor nodes within its range. Thus, the establishment of trust among sensors in WSNs is of utmost significance in achieving secure and dependable communication while preventing and mitigating potential security threats [10]. To achieve this, a trust management framework [11] can be built where one sensor evaluates the trustworthiness of another sensor node.

The existing body of literature offers diverse solutions for tackling the issues encountered by sensors within the domain of WSNs. However, many research studies depend on encryption and cryptographic approaches [12]. It is important to note that if a sensor is breached, the encryption keys linked to that node will also become compromised. Consequently, relying solely on cryptographic methods cannot effectively identify a sensor node as malicious or benign. Furthermore, cryptography mechanisms exhaust a substantial quantity of resources, such as battery life and memory, rendering them ineffective for sensors with limited resources in WSNs. In response to these challenges, trust evaluation [5] has arisen as a broadly recognized method to improve the robustness and security of WSNs. A further layer of security [13] is added by the management of trust, which enables trustors (trust evaluators) to deal with the uncertainties brought on by the unpredictable actions of other WSN participants (trustees). The role of the trustors is to assess the trustworthiness of the trustees. While the academic literature contains numerous trust management models concentrated on trust calculation within WSNs [14], researchers have encounter difficulties when attempting to compare these models with current approaches. Hence, there is a need for a detailed, analytical, and comprehensive comparison of trust management models for WSNs. This comprehensive comparison will serve as a foundational framework for academics to facilitate precise comparisons between proposed trust evaluation frameworks and existing models.

In this research paper, we present a comparative assessment of these three trust evaluation frameworks designed specifically for WSNs: “Lightweight Trust Management based on Bayesian and Entropy (LTMBE) [15]”, “Beta-based Trust and Reputation Evaluation System (BTRES) [16]”, and “Lightweight and Dependable Trust System (LDTS) [17]”. To conduct a comprehensive comparative analysis of trust management models for WSNs, it is imperative to establish a common framework for comparison. All three trust management models require the same type of input data to evaluate trust, i.e., successful and failed interactions between the sensor nodes. Secondly, all three models evaluate communication-based trust that involves direct trust, indirect trust, and overall trust between the sensor nodes. The differentiating features between these three trust models are trust evaluation criteria and network structure. The recent studies on trust management are highly heterogeneous in nature and require different types of input data and numerous assumptions to conduct a comparative analysis. Thirdly, the major challenge is data availability. None of the recent studies on trust management use publicly available datasets or provide access to their data. The LTMBE, BTRES, and LDTS models contain sufficient similarities and differences to conduct an unbiased comparative analysis. We overcome various challenges of these trust evaluation frameworks for WSNs, such as the scarcity of accessible datasets and a clear interpretation of trust management schemes by providing a detailed comparative analysis and making certain assumptions. These assumptions are necessary to provide a common and consistent background to compare these three models as they require common, but also different, information and data. We also assess the effectiveness and findings of these models by performing extensive experiments under different scenarios. Moreover, we also experimentally evaluate and compare the performance of these three trust evaluation frameworks against two cyber-attacks: (1) an on–off attack and (2) a bad-mouthing attack. The results obtained from this comparative evaluation can act as a foundational framework for future research on trust evaluation frameworks, enabling academics or researchers to conduct effective comparisons with their own proposed trust evaluation models. To our knowledge, we are the first to perform this type of analysis. The purpose is to assess the effectiveness and resiliency of these trust models against these common sensor attacks.

In summary, the contributions of this paper are as follows:

1. Comprehensive comparative analysis of three trust management models: LTMBE, BTRES, and LDTS.
2. Establishing network configurations and assumptions of the LTMBE, BTRES, and LDTS schemes for an unbiased experimental analysis and evaluation against an on–off attack and a bad-mouthing attack.
3. Experimental performance evaluation and comparison of the LTMBE, BTRES, and LDTS models against an on–off attack and a bad-mouthing attack.

## 2. Background and Related Work

In recent years, significant investigation has been carried out on trust management in WSNs, leading to a substantial volume of literature dedicated to this subject.

This section first describes the fundamental concepts of trust management in WSNs such as trust, direct trust, indirect trust, and recommender sensor nodes followed by an overview of the existing research literature on trust management models for WSNs.

### 2.1. Trust

**Figure 1:**
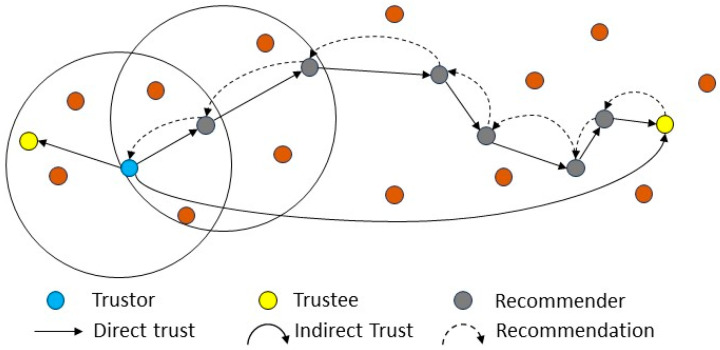
Direct and indirect trust.

In the literature, trust has been defined in various ways, encompassing factors such as risk, reliability, quality of services, accessibility, and utility, among others. Here, “trust” pertains to the level of certainty or assurance that a device possesses regarding another device’s capability to carry out its designated task, derived from their previous communications and observed performance [18]. In the case of WSNs, the trust value is essentially employed to indicate whether or not a sensor is willing and able to operate normally. Figure 1 shows the different trust relationships, such as direct trust, indirect trust, and how the trustor communicates with the trustee. The red nodes in Figure 1 denotes common sensor nodes that are not participating in the trust evaluation process and the black circles denote the communication range of the sensor nodes.

### 2.2. Direct Trust

When trust is determined through the evaluation of direct interactions and observed behaviors, it is referred to as a direct trust score [19]. This measure serves to indicate the level of trust that exists between two neighboring nodes.

### 2.3. Indirect Trust

An indirect trust may be established in situations where a trustor node is unable to directly observe the communication behaviors of a trustee node. The indirect trust score is determined by the recommendations offered by other sensors. To evaluate the indirect trust, the trustor sends out a request to its nearest direct recommender as shown in Figure 1. This request is directly forwarded to other recommenders until it reaches the trustee node. The trustee, in response, sends its information in the form of feedback via the recommenders until the feedback is delivered to the trustor for indirect trust evaluation.

### 2.4. Properties of Trust

Trust can be characterized by three primary properties: composability, transitivity, and asymmetry [20]. Asymmetry denotes that if sensor A places trust in sensor B, it does not inherently imply that sensor B reciprocates the trust in sensor A. The property of transitivity indicates that the trust values have the potential to be propagated through a sequence of trusted nodes. For instance, when sensor A places trust in sensor B, and sensor B has trust in sensor C, it follows that sensor A, to some extent, trusts sensor C. This specific property holds significant relevance in trust calculations involving nodes that are not directly adjacent to each other. Composability refers to the ability to combine trust values obtained from multiple paths to arrive at an integrated value.

### 2.5. Literature Review

Khan et al. [21] propose an innovative and all-encompassing trust evaluation method for extensive WSNs known as LTS. The proposed framework uses clustering to enhance security, trustworthiness, and cooperation by detecting malicious or faulty sensors. LTS contains two primary forms of communication: intra-cluster communication and inter-cluster communication. The WSN topology presented in this approach contains three main components such as (1) cluster members, (2) cluster heads, and (3) a base station. Functioning as an aggregator node, the base station monitors the overall communication between cluster heads and their respective cluster members. The innovation in this approach lies in its utilization of two adjustable features, namely, punishment and trust severity, allowing adjustments based on the specific requirements of the application. To make communication easy, the proposed approach generates a unique identity for each sensor and secures the device against external attacks. Simulations and theoretical analysis are performed to assess the proposed technique in terms of trust score, the cost required to compute trust, communication overheads, and detection of malicious sensors. This model establishes a secure channel by using a key management scheme. However, there are no details as to how the keys are managed and exchanged in this channel.

Zhao et al. [22] propose an efficient “exponential-based trust and reputation evaluation system (ETRES)”. The presented scheme applies the “exponential distribution” to derive the expression of trust and reputation of sensor nodes. Additionally, entropy theory is used by the proposed scheme to address the degree of uncertainty. In cases where the degree of uncertainty significantly increases, ETRES computes both indirect trust and direct trust to augment the credibility of the final trust score. Due to this criterion, computation power and energy are saved, which can prolong the life of the sensor nodes. Furthermore, the proposed trust model is evaluated to test its effectiveness in mitigating various cyber-attacks including on–off attacks, collusion attacks, selective forwarding attacks, and slander attacks. The evaluations are conducted by performing simulations with four different scenarios and comparing the results with other existing trust management models. The ETRES scheme is evaluated in terms of how much energy of the nodes is saved during trust evaluation as compared to other models. However, the model does not contain any metrics or equations to evaluate the energy of the sensor nodes.

Zheng et al. [23] propose a dynamic network security mechanism to ensure reliability and quickly detect malicious nodes. The WSN topology presented in this approach contains four types of nodes, namely, monitoring nodes, ordinary nodes, base stations, and domain management nodes. The ordinary nodes sense and collect data and calculate direct and indirect trust, whereas the domain management nodes are a subset of ordinary nodes. These nodes have high trust scores and ensure that the nodes within a domain are secure by isolating the malicious nodes. The monitoring nodes interact with the base station and continuously observe the conduct of the domain management nodes. Upon receiving reports from the monitoring nodes, the base station nodes are tasked with selecting and overseeing the domain management nodes. The direct trust score is evaluated by using the direct interactions between the nodes and employing the beta distribution. On the other hand, the indirect trustworthiness is determined by considering recommendations from the nodes that interact with both the trustor and the trustee. To assess the proposed trust management system, simulations are performed along with security analysis to show how the computed trust scores can detect malicious nodes within the network. This scheme lacks a threat model and a security analysis to show how the proposed model performs against different cyber-attacks.

Meng et al. [24] propose a trust evaluation model that integrates trust management based on Bayesian principles with wireless traffic sampling, specifically designed for intrusion detection applications. The proposed intrusion detection system based on trust can assess the trust score of a sensor by considering its packet status within a hierarchical WSN. Additionally, it identifies malicious sensors by selecting a suitable trust threshold. The hierarchical WSN topology presented in this approach contains cluster heads, sensors, and a base station, where the sensors gather packet status, the cluster heads compute trust scores for all the sensors, and the base station is a central authority that is assumed to be fully trusted. The proposed approach is evaluated against inside attacks such as betrayal attacks by performing two major experiments in a real network and simulated environments. The objective of the first experiment is to study the effect of the trust threshold by conducting a betrayal attack and flooding the simulated WSN with malicious packets. The second experiment is performed in a real network environment. The experimental results conclude that a threshold of 0.8 is ideal for detecting malicious nodes quickly. The model uses static threshold selection for trust evaluation and does not suggest how the threshold can be varied for different network scenarios or WSN applications.

Hajar et al. [25] propose a lightweight trust management scheme (LTMS) for “Wireless Medical Sensor Networks (WMSNs)”. The proposed scheme evaluates trust for different types of sensor nodes such as in-body, on-body or wearables, off-body or near-body sensor nodes, and sink nodes. The sink node is an aggregator node that connects a Body Sensor Network (BSN) to other BSNs or the internet. The in-body sensor nodes are devices that are implanted inside the human body to check vital signs such as pacemakers. The wearables and near-body sensor nodes are placed on the body’s surface or in proximity to it. The proposed LTMS method provides security against packet drop attacks launched by malicious nodes. The trust evaluation scheme of the LTMS uses beta distribution and thresholds are introduced to categorize trust scores to detect if a sensor node is malicious or benign. Furthermore, simulations are performed to evaluate the LTMS, and the outcomes demonstrate that the LTMS can effectively defend and mitigate complex on–off attacks, significantly reducing computation overhead. The LTMS model is only evaluated against one cyber-attack, i.e., on–off attack, and does not consider the possibility of malicious nodes/recommenders that provide false data to frame honest nodes.

Khan and Singh [26] present a trust evaluation framework for WSNs that is both efficient and precise, focusing on essential requirements such as resource optimization and enhancing security. The trust framework presented in this research calculates trust with greater precision by incorporating both communication data and sensor data, surpassing the accuracy of other trust management frameworks such as LDTS, ADTC, and LWTM. The presented trust management model incorporates certain features such as “authentication-based data trust”, optimal task scheduling, and minimum reward and punishment features for dishonest nodes. These features improve security by detecting and mitigating internal attacks. The WSN topology employed in this method is a cluster-based WSN with two types of entities: cluster members and cluster heads. The trust evaluation process of this scheme begins with the computation of communication trust and data trust which uses input data such as the number of failed and successful communications. The trust values are within the range of [0, 4] with different thresholds to categorize a sensor node based on its trust score. Once the communication and data trust are calculated, the next step is to calculate the “authentication-based data trust” and “scheduler-based node trust”. Finally, simulations are performed to assess the proposed model with two different scenarios. The trust score of the proposed model ranges from 0 to 4, which is not broad enough to accurately identify whether a sensor node is malicious or normal. Furthermore, the trust model severely penalized bad behavior of the sensor nodes which even lowers the trust score of the normal nodes.

Labraoui et al. [27] propose a generic trust evaluation model employing risk and reputation to assess communication trust in WSNs. The proposed framework evaluates risks to address potential sensor malfunction, making the proposed model resistant to on–off attacks. There are two types of evaluations in the proposed model, namely risk evaluation and reputation evaluation which is then combined to form an overall trust score. The assessment of reputation relies on both direct and indirect communications among the sensor nodes, providing an overall evaluation of the consistent behavior of these nodes, while risk assessment is grounded in the information obtained from interactions between sensors. All the trust scores computed in this method are calculated within a time window. Specifically, the sliding window method is used with varying time units where the proposed trust evaluation scheme computes trust for each time unit and forgets past computations as new units are added. The proposed model specifically computes the risk score by using certain communication-based metrics such as successful interactions, unsuccessful interactions, and the ratio of the two terms. Finally, after calculating the overall trust score, a sensor is classified into three categories such as trusted, malicious, or uncertain by establishing thresholds for each category. The proposed model fails to offer a solution for handling the uncertainty surrounding the trustworthiness of the sensors, nor does it present a method for quantifying or mitigating the uncertainty in evaluating the trust score.

Fang et al. [28] propose a trust management model for Fog computing Industrial Wireless Sensor Networks (F-IWSNs) known as “GDTMS” that is based on Gaussian distribution. First, the direct trust score in this model is assessed between the trustor and the trustee by analyzing the historical direct interactions between them. These interactions are categorized into two types such as successful and failed interactions which are assumed to obey the Gaussian distribution. Subsequently, the assessment of indirect trust involves utilizing recommendations from the recommending nodes. The integrated trust value is then obtained by merging the direct and indirect trust values. Once the integrated trust is obtained, grey decision making is used to select the most reliable sensor node for packet forwarding. The GDTMS model introduces potential cyber-attacks theoretically; however, it lacks a comprehensive security analysis, and no attacks have been demonstrated in the experimental evaluation of the model.

Bhargava et al. [29] proposed the “Dempster Shafer Theory (DST)-based edge-centric IoT-specific trust model (DEIT)” for WSNs. The DEIT mechanism enables edge servers to compute the trustworthiness of edge devices based on their behavioral metrics such as message drop rate and message modification rate. The edge devices are assessed with certain variables such as the credibility variable, re-compensating variable, degradation variable, pardoning variable, and punishment variable. These variables either increase or decrease the trust score of an edge device based on the behavior of the device and the nature of variable. This classification of edge devices as honest or malicious is used to compute both direct and indirect trust scores, which are combined using the DST rule of combination to form an integrated trust score. The DEIT model proposes DST as a solution to address the uncertainty in the trust evaluation; however, it lacks a security analysis showing how well the model performs against cyber-attacks.

Luo et al. [30] propose a trust management model for cluster-based WSNs which evaluates the trust scores based on the behavior of the sensor nodes. The proposed model uses a hash algorithm for creating labels of the sensor nodes to differentiate between the normal nodes and attacker nodes. The WSN structure of the model contains a management center and different clusters. The management center acts as a base station for all the clusters in the network. The trust score is evaluated using the beta density function and the model is experimentally evaluated by performing simulations evaluating how the trust score changes with time. The proposed model uses the SHA-1 hashing algorithm to generate a unique fingerprint of the sensor nodes. However, SHA-1 is vulnerable to collision attacks and its security weakens over time as the computational power increases.

Zhang et al. [31] propose a trust management scheme that uses DST to detect malicious nodes. The trust model first considers the spatio-temporal correlations of the sensor data to calculate the trust degree of the sensor nodes. Next, the model uses DST to count the number of interactions with respect to three categories, i.e., trust, distrust, and uncertainty, to evaluate the direct and indirect trust score between the sensor nodes. Finally, a flexible synthesis method is used to calculate an integrated trust score to detect malicious nodes. The proposed model is experimentally evaluated by performing simulations with varying simulation parameters. The simulation results show the change in trust score with time and malicious node detection rate with time. The proposed model provides a solution to quantify and mitigate the uncertainty in the trust evaluation process by introducing DST. However, a threat model and a security analysis are lacking as a trust management model must demonstrate its performance in terms of security against cyber-attacks.

We specifically selected the LTMBE, BTRES, and LDTS models for trust evaluation and comparison because they all utilize the same type of communication data. These data comprise successful and unsuccessful interactions between the trustor and trustee, forming the basis for evaluating communication trust. Notably, LTMBE and BTRES share similar direct trust evaluation criteria and network topologies, as both models employ beta distribution. Additionally, acquiring an open-source communication dataset for trust evaluation poses a significant challenge; hence, we utilized the sample dataset from the LTMBE model to conduct an unbiased analysis, comparison, and evaluation of all three models.

## 3. Methodology

This section outlines the network configurations and the trust evaluation process for the three trust management frameworks designed for WSNs: “LTMBE”, “BTRES”, and “LDTS”. We specifically choose these three trust management models for various reasons, mainly due to their prominence in the literature and the detailed depth to develop a comparative scenario. Additionally, in all these models the criteria for trust evaluation rely on communication data such as the number of successful and unsuccessful interactions. Moreover, these three models cover a broad variety of WSN topologies such as cluster-based WSNs, single-hop and multi-hop networks, and various trust evaluation relationships.

### 3.1. Trust Evaluation of LTMBE

In the LTMBE framework [15], the primary goal is to keep the trust assessment scheme lightweight. This scheme was made lightweight by incorporating recommendations and relying on indirect trust solely when the direct trustworthiness fell under a specified limit. Consider a case where sensor i is assessing the trustworthiness of sensor j, the criteria for this trust evaluation are explained in the following way:

#### 3.1.1. Direct Trust Evaluation

**Figure 2:**
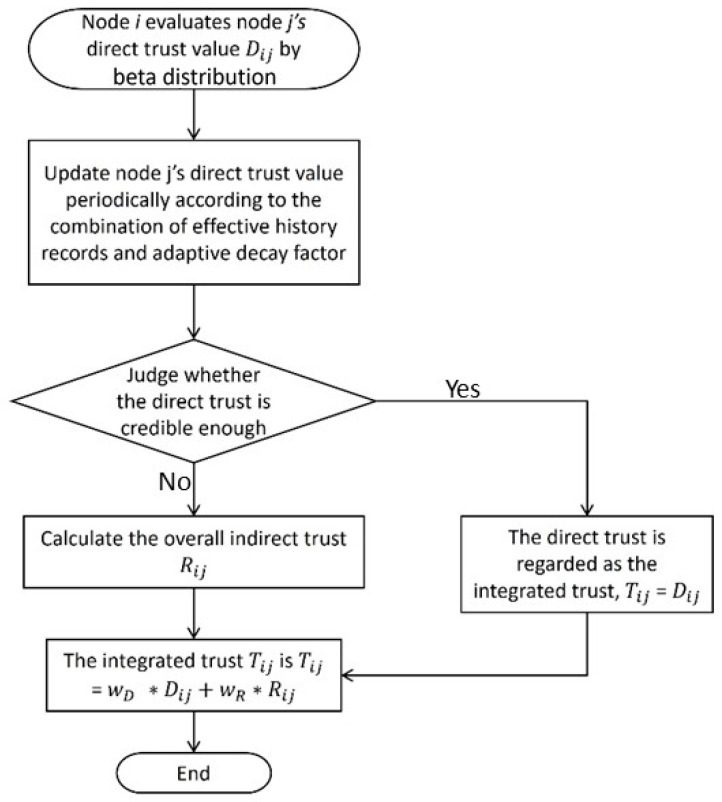
LTMBE trust assessment criteria.

In the LTMBE scheme, a prior probability distribution known as beta distribution is utilized for establishing direct trust involving sensor i and sensor j. The fundamental parameters capturing the direct interactions between sensor i and sensor j include the cumulative count of prior successful communications, denoted as $\alpha_{ij}$, and the cumulative count of prior failed communications, represented as $\beta_{ij}$. Figure 2 depicts the trust assessment process employed in the LTMBE scheme. The direct trust score, $D_{ij}$ among sensor i and sensor j is computed as follows:

(1) $$D_{ij}=\frac{\alpha_{ij}+1}{\alpha_{ij}+\beta_{ij}+1}$$

#### 3.1.2. Direct Trust Update

Consider that sensor i establishes its starting value of direct trust at time t=0 and then updates it after a given interval of time t. In this scheme, only relevant history data are stored instead of retaining all records to conserve the memory of nodes and enhance dynamic adaptability. The temporal span t is partitioned into h segments. Within each segment, sensor i assesses the behavior of sensor j, observing $\alpha_{ij}(k)$ and $\beta_{ij}(k)$ which denote the successful and failed communications, respectively, where k=0, t/h, 2t/h, 3t/h, …, (h−1)t/h.

Subsequently, the direct trustworthiness score is adjusted based on the relevant past information. Let s equal 0 if the behavior of sensor node j is considered malicious and s equal 1 if the behavior is deemed normal.

In the LTMBE scheme, a parameter known as “adaptive decay factor (λ)” is established to account for the reliability of the historical data which diminish over time. Additionally, this feature can dynamically set weights to the corresponding data.

(2) $$\lambda =\left\{\begin{array}{c} \lambda_{h},\;\;\;\;\;\;\;s=1\\ \lambda_{l},\;\;\;\;\;\;\;s=0 \end{array}\right.$$

where $0<\lambda_{l}<\lambda_{h}<1$. A node’s behavior can be identified as normal if the cumulative count of successful communications is greater than failed ones. In this case, s becomes 1 and $\lambda =\lambda_{h}$. However, if a node’s behavior is identified as malicious then s=0 and $\lambda =\lambda_{l}$. The adaptive decay factor is a weight that rewards honest behavior by significantly increasing the trustworthiness between two sensors and punishes malicious behavior by rapidly decreasing the trustworthiness between two sensors when there is malicious activity. The following equations show how to compute the direct trust score at time t:

(3) $$D_{ij}\left(t\right)=\frac{\alpha_{ij}\left(t\right)+1}{\alpha_{ij}\left(t\right)+\beta_{ij}\left(t\right)+1}$$

(4) $$\alpha_{ij}\left(t\right)=\frac{\sum_{k=0}^{k=\frac{\left(h-1\right)t}{h}}\lambda^{\frac{\left(h-1\right)t}{h-k}}\alpha_{ij}\left(k\right)}{\sum_{k=0}^{k=\frac{\left(h-1\right)t}{h}}\lambda^{\frac{\left(h-1\right)t}{h-k}}}+s$$

(5) $$\beta_{ij}\left(t\right)=\frac{\sum_{k=0}^{k=\frac{\left(h-1\right)t}{h}}\lambda^{\frac{\left(h-1\right)t}{h-k}}\beta_{ij}\left(k\right)}{\sum_{k=0}^{k=\frac{\left(h-1\right)t}{h}}\lambda^{\frac{\left(h-1\right)t}{h-k}}}+\left(1-s\right)$$

#### 3.1.3. Direct Trust Threshold

Many trust evaluation frameworks compute both direct trust and indirect trust to compute the final trustworthiness score. However, to conserve the memory of the sensors and maintain the lightweight nature of the trust evaluation scheme, LTMBE computes the indirect trust score only when the direct trustworthiness is under a certain limit. This limit or threshold is known as the confidence level γ, which is calculated in the following way:

(6) $$\gamma =\frac{\int_{D_{ij}-\varepsilon }^{D_{ij}+\varepsilon }p^{\alpha_{ij}-1}{\left(1-p\right)}^{\beta_{ij}-1}dp}{\int_{0}^{1}p^{\alpha_{ij}-1}{\left(1-p\right)}^{\beta_{ij}-1}dp}$$

where ε ranges from 0 to $min(1-D_{ij},\;D_{ij})$ and represents the error level. Since the maximum trust value this scheme can achieve is 1, we set the error level ranging from 0 to a minimum of either value $D_{ij}$ or $\left(1-D_{ij}\right)$, whichever is minimum. Consider that $\gamma_{0}$ is the confidence level threshold which lies within the range of 0.8 to 1. If $\gamma \geq \gamma_{0}$, then $D_{ij}$ is considered as the final trust score; otherwise, the indirect trust score needs to be calculated. This limit can be adjusted based on the specific WSN application in use. For applications that require high security [32], this threshold can be set at a higher limit.

#### 3.1.4. Indirect Trust Evaluation

To calculate the indirect trust score, entropy theory is employed to first calculate the weights of the recommendations. The entropy of a recommendation R is calculated as follows:

(7) $$H\left(R\right)=-R{log}_{2}R-\left(1-R\right){log}_{2}\left(1-R\right)$$

**Figure 3:**
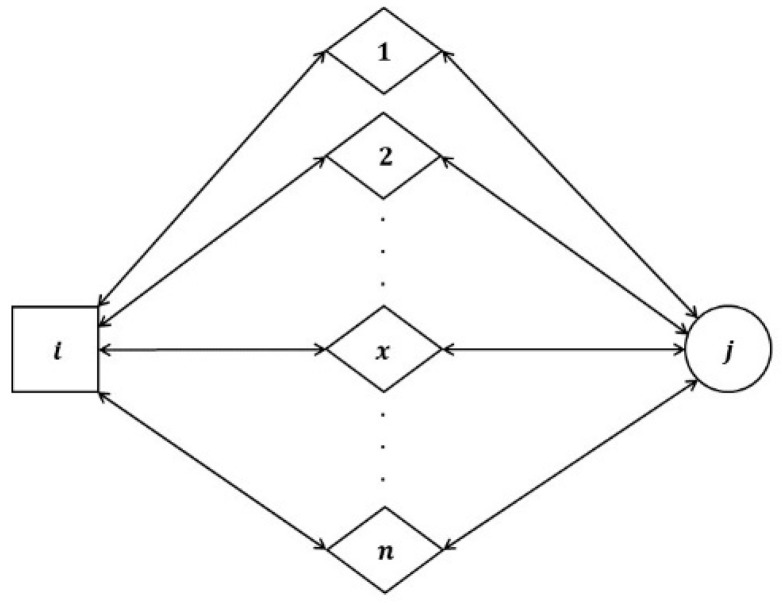
LTMBE indirect trust configuration.

Consider a scenario where sensor node i sends out a signal to its neighboring sensor nodes, seeking recommendations for sensor node j as depicted in Figure 3. Once the recommenders, which are the common sensors among sensor i and sensor j, receive the signal they respond to sensor node i with the recommendation.

Consider a case where a recommender node x transmits its direct communication data $\left(\alpha_{xj},\beta_{xj}\right)$ regarding sensor node j back to sensor i. Subsequently, sensor i integrates the recommendation information received from recommender x with its communication data $\left(\alpha_{ix},\beta_{ix}\right)$ related to recommender x to compute the indirect trust score $R_{ij}^{x}$ in the following way:

(8) $$R^{x}\alpha_{ij}=\frac{2*\alpha_{ij}\alpha_{xj}}{\left(\beta_{ix}+2\right)*\left(\alpha_{xj}+\beta_{xj}+2\right)+(2*\alpha_{ix})}$$

(9) $$R^{x}\beta_{ij}=\frac{2*\alpha_{ix}\beta_{xj}}{\left(\beta_{ix}+2\right)*\left(\alpha_{xj}+\beta_{xj}+2\right)+(2*\alpha_{ix})}$$

(10) $$R_{ij}^{x}=\frac{R^{x}\alpha_{ij}+1}{R^{x}\alpha_{ij}+R^{x}\beta_{ij}+2}$$

Next, we compute the entropy and the weights of these recommendations. Entropy is a principle which is used in information theory, thermodynamics, and statistical mechanics. The fundamental idea revolves around quantifying the degree of randomness present in a signal or a random event. Since trust is recognized as an uncertain event, it can be assessed using entropy. The entropy of the recommendation $R_{ij}^{x}$ can be calculated as follows:

(11) $$H\left(R_{ij}^{x}\right)=-R_{ij}^{x}{log}_{2}R_{ij}^{x}-\left(1-R_{ij}^{x}\right){log}_{2}\left(1-R_{ij}^{x}\right)$$

Next, we calculate the weight $w_{x}$ of this recommendation using entropy theory:

(12) $$w_{x}=\frac{1-\frac{H(R_{ij}^{x})}{{log}_{2}R_{ij}^{x}}}{\sum_{x=1}^{x=n}\left(1-\frac{H(R_{ij}^{x})}{{log}_{2}R_{ij}^{x}}\right)}$$

(13) $$R_{ij}=\sum_{x=1}^{x=n}\left(w_{x}*R_{ij}^{x}\right)$$

#### 3.1.5. Final Trust Score Evaluation

The final trust score can be calculated in the following way:

(14) $$\left\{\begin{array}{l} T_{ij}=D_{ij},\;\;\;\;\;\;\;\;\;\;\;\;\;\;\;\;\;\;\;\;\;\;\;\;\;\;\gamma \geq \gamma_{0}\\ T_{ij}=w_{D}*D_{ij}+w_{R}*R_{ij},\;\;\;\;else \end{array}\right.$$

where $w_{D}$ and $w_{R}$ are computed as follows:

(15) $$w_{D}=\frac{1-\frac{H(D_{ij})}{{log}_{2}D_{ij}}}{\left(1-\frac{H(D_{ij})}{{log}_{2}D_{ij}}\right)+\left(1-\frac{H(R_{ij})}{{log}_{2}R_{ij}}\right)}$$

(16) $$w_{R}=\frac{1-\frac{H(R_{ij})}{{log}_{2}R_{ij}}}{\left(1-\frac{H(D_{ij})}{{log}_{2}D_{ij}}\right)+\left(1-\frac{H(R_{ij})}{{log}_{2}R_{ij}}\right)}$$

For the above Equations (15) and (16), the entropy of the direct trust $H(D_{ij})$ and indirect trust $H(R_{ij})$ can be calculated as follows:

(17) $$H\left(D_{ij}\right)=-D_{ij}{log}_{2}D_{ij}-\left(1-D_{ij}\right){log}_{2}\left(1-D_{ij}\right)$$

(18) $$H\left(R_{ij}\right)=-R_{ij}{log}_{2}R_{ij}-\left(1-R_{ij}\right){log}_{2}\left(1-R_{ij}\right)$$

Overall, the LTMBE trust evaluation scheme is a lightweight scheme to evaluate trust by using the beta distribution function and entropy theory. This scheme uses effective historical data, instead of considering the whole data to update the trustworthiness score of nodes. This saves memory and reduces power consumption. Moreover, this scheme exclusively computes indirect trust when the direct trust is beneath the confidence level limit, rendering it energy-efficient.

### 3.2. Trust Evaluation of BTRES

In this section, we show how the trust is assessed in the BTRES scheme [16]. In this scheme, both direct trust and indirect trust are calculated, unlike the LTMBE scheme which only calculates indirect trust if the direct trust falls below a certain threshold.

#### 3.2.1. Trust Assessment Criteria

**Figure 4:**
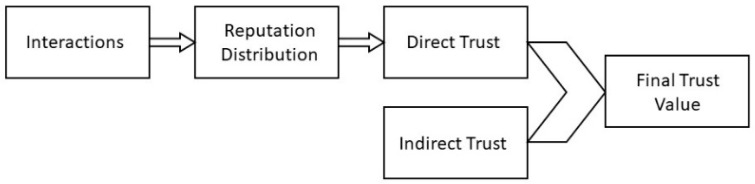
BTRES trust assessment criteria.

Before proceeding with any interaction, sensor i assesses the trust of sensor j when they initiate communication. The evaluation criteria are depicted in Figure 4. Initially, by performing an analysis of the past communications, sensor i determines the reputation of sensor j. Afterward, sensor i calculates sensor j’s direct trustworthiness value which ranges from 0 to 1 and is based on the distribution of reputation data. Next, the indirect trustworthiness value of sensor j is then obtained by sensor i by requesting information from its neighbor nodes which is subsequently incorporated into the overall trust calculation.

#### 3.2.2. Reputation and Direct Trust

For calculating reputation $R_{ij}$, a beta distribution is utilized, incorporating two parameters, namely, ‘a’ and ‘b’. Here, ‘a’ corresponds to the successful communications, while ‘b’ accounts for the failed interactions between sensor i and sensor j. To calculate the direct trust ${DT}_{ij}$, the probabilistic estimate of the reputation is used.

(19) $$R_{ij}=Beta\left(a+1,\;b+1\right)$$

(20) $${DT}_{ij}=E\left(R_{ij}\right)=\frac{a+1}{a+b+2}$$

#### 3.2.3. Reputation Update and Aging

As the number of interactions between sensor i and sensor j increases, the reputation and communication data will be updated. Therefore, the updated interactions between sensor i and j can be denoted as follows:

(21) $$a_{new}=a+r$$

(22) $$b_{new}=b+s$$

#### 3.2.4. Indirect Trust Evaluation

Assume that sensor node i can assess the trustworthiness of m neighboring nodes, and sensor node i obtains an indirect trust assessment of sensor node j through these nodes. Let $\left(a_{j}^{x},\;b_{j}^{x}\right)$ represent the indirect communication data of sensor x with sensor j. Sensor node i already possesses the communication data of sensor node j and other neighboring nodes; for instance, neighbor node x is denoted as $\left(a_{j},\;b_{j}\right)$ and $\left(a_{x},\;b_{x}\right)$. The final trust or the integrated value $T_{ij}$ between sensor i and sensor j can be evaluated in the following way:

(23) $$T_{ij}=\alpha .{DT}_{ij}+\sum_{x=1}^{m}\beta_{x}.{IT}_{xj}=\alpha .{DT}_{ij}+\beta .\sum_{x=1}^{m}\gamma_{x}$$

(24) $$T_{ij}=\alpha .\frac{a_{j}+1}{a_{j}+b_{j}+2}+\beta .\sum_{x=1}^{m}\gamma_{x}.\frac{a_{j}^{x}+1}{a_{j}^{x}+b_{j}^{x}+2}$$

As illustrated in Equation (24), a sensor node’s integrated trust score consists of both indirect and direct trust. The term ${IT}_{xj}$ in Equation (23) denotes the indirect trust score of sensor x to sensor j where m denotes the count of neighbor nodes. In Equations (23) and (24), the weight values of the direct and indirect trust evaluations are represented by parameters α and β, respectively, where α+β=1. Additionally, the weight of every recommender or neighbor node is denoted by $\gamma_{x}$ and $\sum_{x=1}^{m}\beta_{x}.{IT}_{xj}$ denotes the sum of the indirect evaluations of the recommender nodes. The recommender weight $\gamma_{x}$ can be calculated as follows:

(25) $$\gamma_{x}=\frac{{DT}_{ix}}{\sum_{x=1}^{m}{DT}_{ix}}=\frac{\frac{a_{x}+1}{a_{x}+b_{x}+2}}{\sum_{x=1}^{m}\frac{a_{x}+1}{a_{x}+b_{x}+2}}$$

The BTRES scheme is similar to the LTMBE scheme based on how it calculates direct trust by using the beta distribution function. However, unlike the LTMBE scheme, the BTRES scheme is not energy-efficient as it calculates both direct and indirect trust for every pair of sensor nodes and finally fuses the two types of trust to obtain an integrated trust value.

### 3.3. Trust Evaluation of LDTS

To evaluate the trustworthiness between nodes, this scheme employs a cluster-based WSN, unlike the LTMBE and BTRES schemes that requires a sensor network containing clusters where the data are reported to a central authority known as a base station.

#### 3.3.1. Network Structure and Hypotheses

There are two categories of sensor nodes: “cluster members (CMs)” and “cluster heads (CHs)” that make up the WSN of the LDTS scheme [17]. CMs within a given cluster can communicate directly with their corresponding CHs. On the other hand, CHs have the capability to relay the gathered data to the base station (BS) through other CHs. It is presumed that an established clustering method, like the ones outlined in [33], is applied to structure the nodes or devices into clusters. The LDTS scheme uses a cluster-based WSN since cluster-based topologies have high energy efficiency due to the aggregation and data forwarding between CHs and the BS. This reduces the number of sensor nodes connecting to the BS. Moreover, cluster-based WSNs prolong network lifetime [34].

#### 3.3.2. Trust Evaluation

The LDTS scheme utilizes two primary levels of trust: “inter-cluster” trust, which pertains to trust between different clusters, and “intra-cluster” trust, which relates to trust within the same cluster. Trust within the same cluster comprises two specific trust relations: the direct trust among cluster members and the indirect trust from cluster heads to cluster members. Likewise, trust between clusters includes two types of relations: direct trust among cluster heads and indirect trust from the base station to cluster heads.

#### 3.3.3. Intra-Cluster Trust Evaluation

1. CM-CM Direct Trust

A CM uses direct observations such as the total count of unsuccessful and successful interactions to compute the direct trust of its neighboring CMs. Furthermore, all CMs communicate in bidirectional wireless channels and calculate the direct trust value in the following way:

(26) $$D_{x,\;y}\left(\Delta t\right)=\left(\frac{a_{x,\;y}\left(\Delta t\right)}{a_{x,\;y}\left(\Delta t\right)+f_{x,\;y}\left(\Delta t\right)}\right)\left(\frac{1}{\sqrt{f_{x,\;y}\left(\Delta t\right)}}\right)$$

where $D_{x,\;y}$ is the direct trust between CM x and CM y and Δt is the time window which can be varied according to the application or network scenario. $a_{x,\;y}\left(\Delta t\right)$ and $f_{x,\;y}\left(\Delta t\right)$ are the total number of successful and failed communications between CM x and CM y during time $\left(\Delta t\right)$.

2. CH-CM Feedback Trust

Suppose there are a total of (k−1) CMs in a cluster where a request packet will be regularly transmitted throughout the cluster by a CH. As a result, every CM within the cluster will relay their trust scores related to other CMs, toward the CH. Once the CH receives the trust scores from the requested CMs, that CH will store all the data in a matrix H as follows:

(27) $$H=\left(\begin{array}{cccc} D_{1,\;1} & D_{1,\;2} & \ldots & D_{1,\;k-1}\\ D_{2,\;1} & D_{2,\;2} & \ldots & D_{2,\;k-1}\\ \vdots & \vdots & \ddots & \vdots \\ D_{k-1,\;1} & D_{k-1,\;2} & \ldots & D_{k-1,\;k-1} \end{array}\right)$$

In the matrix H, if x=y, a node’s trust score with itself will be deleted to reduce boasting. These include all the diagonal elements of the matrix H. The CH-CM feedback trust or the indirect trust is calculated by using the beta probability density function as follows:

(28) $$R_{ch,\;y}\left(\Delta t\right)=E\left(\varphi \left(p|s,\;f\right)\right)$$

where $R_{ch,\;y}\left(\Delta t\right)$ is the indirect trust between a CH and a CM y. For the binary events (s, f), where s is the number of positive feedback $\left(D_{x,\;y}\geq 0.5\right)$ and f is the number of negative feedback $\left(D_{x,\;y}<0.5\right)$, p represents the posteriori probability of these events. The expression, $E\left(\varphi \left(p|s,\;f\right)\right)$ represents the statistical expectation and is computed as follows:

(29) $$E\left(\varphi \left(p|r,\;v\right)\right)=\frac{s+1}{s+f+2}$$

#### 3.3.4. Inter-Cluster Trust Evaluation

1. CH-CH Direct Trust

CHs serve as a virtual backbone for inter-cluster routing in cluster-based WSNs, allowing them to transmit aggregated data via other CHs to the base station. Similar to the direct trust calculation among CMs, a CH calculates direct trustworthiness value with its neighboring CH by using direct observations such as the total count of failed and successful interactions with its neighbor. The CH-CH direct trust can be calculated in the following way:

(30) $${CT}_{i,\;j}\left(\Delta t\right)=\left(\frac{A_{i,\;j}\left(\Delta t\right)}{A_{i,\;j}\left(\Delta t\right)+F_{i,\;j}\left(\Delta t\right)}\right)\left(\frac{1}{\sqrt{F_{i,\;j}\left(\Delta t\right)}}\right)$$

where ${CT}_{i,\;j}\left(\Delta t\right)$ is the direct trust score between two CHs i and j. $A_{i,\;j}\left(\Delta t\right)$ and $F_{i,\;j}\left(\Delta t\right)$ are the successful and failed communications between the same two CHs during time $\left(\Delta t\right)$.

2. BS-CH Direct Trust

Consider that the WSN consists of n CHs with a single central base station. The BS regularly transmits a request packet throughout the WSN. In response, every CH in the WSN will relay the direct trust data with other CHs to the BS. Once the BS receives the trust scores from the requested CHs, the BS will store all the data in a matrix B as follows:

(31) $$B=\left(\begin{array}{cccc} {CT}_{1,\;1} & {CT}_{1,\;2} & \ldots & {CT}_{1,\;n}\\ {CT}_{2,\;1} & {CT}_{2,\;2} & \ldots & {CT}_{2,\;n}\\ \vdots & \vdots & \ddots & \vdots \\ {CT}_{n,\;1} & {CT}_{n,\;2} & \ldots & {CT}_{n,\;n} \end{array}\right)$$

Similar to matrix H, the CH-CH direct trust values in matrix B that include a CH’s trust score with itself will be discarded to reduce boasting. Therefore, all the diagonal elements of matrix B will be discarded. Beta distribution is employed to calculate the BS-CH indirect trust in the following manner:

(32) $$F_{bs,\;j}\left(\Delta t\right)=\left(\frac{E\left(\varphi \left(p|h,\;l\right)\right)+{CT}_{k,\;j}{\left(\Delta t\right)}^{\prime }}{2}\right)$$

where

$F_{bs,\;j}\left(\Delta t\right)$: BS-CH feedback trust during time Δt;

h, l: number of positive responses ${(CT}_{k,\;j}\geq 5)$ and the number of negative responses ${(CT}_{k,\;j}\leq 5)$, respectively.

${CT}_{k,\;j}{\left(\Delta t\right)}^{\prime }$: Mean value of the combined feedback from (h+l) CHs in the network which is computed as follows:

(33) $${CT}_{k,\;j}{\left(\Delta t\right)}^{\prime }=\frac{\sum_{k=1}^{h+l}{CT}_{k,\;j}\left(\Delta t\right)}{h+l}$$

The LDTS scheme uses a cluster-based WSN unlike the LTMBE and BTRES schemes due to its high energy efficiency and network lifespan. Furthermore, the LDTS scheme uses a strict punishment feature to assess the direct trust score of the sensor nodes. Due to this, the trust value significantly declines even if there is a minute change in the number of failed interactions.

## 4. Results and Analysis

In this section, we present the outcomes of the experimental evaluation conducted on the three trust evaluation frameworks devised for WSNs and offer a comparative analysis to assess the effectiveness of these models.

### 4.1. Experimental Setup

To implement, evaluate, and compare the three trust management models, we conducted experiments using Python on an HP system using Windows 11, equipped with 16 GB RAM and an Intel(R) Core(TM) i7-8565U CPU. Simulated data were employed for the experiments and all three trust management models were evaluated using the same dataset to prevent bias and establish a baseline of comparison. We used simulated data due to a lack of openly available datasets for trust evaluation in the literature.

### 4.2. Assumptions and Scenarios

To facilitate a more comprehensive comparison and analysis of the three trust evaluation frameworks for WSNs, we establish a scenario and introduce specific assumptions for the LTMBE and BTRES schemes. However, the specific assumptions for the LDTS scheme are described in Section 4.3.3 due to its cluster-based WSN topology. We introduce these specific assumptions to establish a common foundation for comparing the three evaluation framework models.

**Figure 5:**
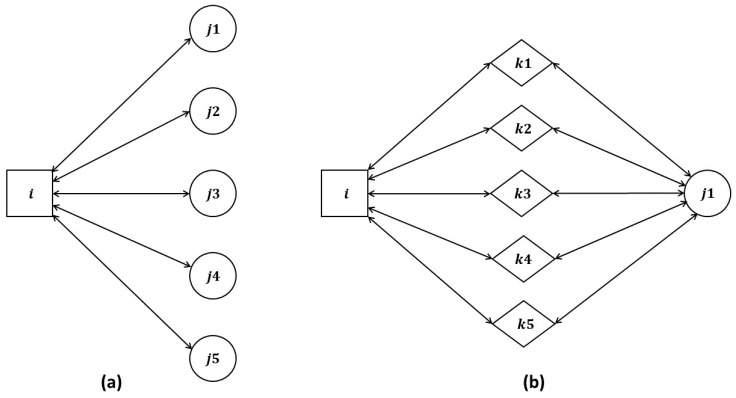
(**a**) Direct trust configuration of node i with nodes j1, j2, j3, j4, and j5 and (**b**) indirect trust configuration with recommenders k1, k2, k3, k4, and k5.

For the LTMBE and BTRES schemes, we consider a case where sensor ‘i’ (the trustor) is engaged in direct communication with five nodes: ‘j1’, ‘j2’, ‘j3’, ‘j4’, and ‘j5’ (the trustees) as shown in Figure 5a. Additionally, there are five recommending nodes denoted as: ‘k1’, ‘k2’, ‘k3’, ‘k4’, and ‘k5’ positioned between node ‘i’ and node ‘j1’ as shown in Figure 5b. Importantly, these five recommending nodes maintain communication links with both node ‘i’ and node ‘j1’.

The general criteria evaluated in all three trust management models encompass the assessment of direct trust between a trustor and the sensor nodes engaged in direct communication. Subsequently, indirect trust, or feedback trust, is computed between a trustor and a trustee by leveraging the communication data provided by the recommending nodes connecting them. These recommending nodes consistently provide feedback about a trustee to its trustor. Ultimately, an integrated or final trust value is determined by synthesizing the direct and indirect trust assessments.

### 4.3. Results

In this section, we present the results of all the three trust management models after implementing them under the specified assumptions as stated in Section 4.2.

#### 4.3.1. LTMBE

To assess the LTMBE scheme, we begin with the communication data, encompassing both successful (α) and unsuccessful (β) communications between node ‘i’ and node ‘j’, as presented in Table 1. We set the error level, ε, to 0.2, a parameter employed in determining the confidence level (γ) within the LTMBE scheme. It is worth noting that this error level falls within the interval $\left(0,\;\min(1-D_{ij},\;D_{ij})\right)$ where $D_{ij}$ represents the direct trust between sensor i and sensor j.

Once we acquire the communication data, we then calculate the direct trust scores and the confidence interval of the 5 nodes j1, j2, j3, j4, and j5 as shown in Table 2.

We have established a threshold, $\gamma_{0}$, set at 0.95 for LTMBE’s confidence interval. As indicated in Table 2, it is evident that $\gamma_{2}>\gamma_{0},\;\gamma_{3}>\gamma_{0},\;\gamma_{4}>\gamma_{0}$ and $\gamma_{5}>\gamma_{0}$. Consequently, following LTMBE’s trust evaluation criteria, we can consider the direct trust values of nodes j2, j3, j4, and j5 as final trust values. Thus, $D_{ij2}=T_{ij2},\;D_{ij3}=T_{ij3},\;D_{ij4}=T_{ij4}$ and $D_{ij5}=T_{ij5}$. These direct trust values are regarded as final trust values due to the confidence level exceeding the threshold $\left(\gamma_{0}\right)$. This approach conserves energy, computational resources, and network capacity, rendering the LTMBE trust evaluation scheme lightweight.

However, in the case of $\gamma_{1}<\gamma_{0}$, node ‘i’ initiates a query command broadcast to the neighbors of node ‘j1’, including nodes k1, k2, k3, k4, and k5, to solicit recommendations about node ‘j1’. Upon receiving the command from node ‘i’, the recommending nodes respond with their recommendations. Subsequently, we calculate the indirect trust for each recommender engaged with node ‘j1’ and proceed to determine the weight for each recommending node using entropy theory. Ultimately, we compute the overall indirect trust of node ‘j1’ based on the obtained weight values, as depicted in Table 3.

To derive the final trust value for node ‘j1’, we initially compute the weights for both direct and indirect trust, followed by their fusion through a weighted sum.

The application of entropy theory for weight distribution in the LTMBE scheme effectively mitigates the influence of malicious recommendations, as demonstrated in Table 3. For instance, when examining the weight assigned to node k4, it becomes evident that the recommendation can be considered malicious.

#### 4.3.2. BTRES

To implement, evaluate, and compare the BTRES scheme with other methods, we utilized the same communication data employed in LTMBE. Within the BTRES scheme, an aging parameter $\left(W_{age}\right)$ is utilized, which assigns greater importance to recent trust scores and communications while diminishing the significance of historical data. We set $W_{age}$ to 0.9, a value that falls within the range of 0 to 1.

Initially, we calculate the direct trust scores between node ‘i’ and nodes j1, j2, j3, j4, and j5, as detailed in Table 4. Similar to the LTMBE scheme, the direct trust scores in BTRES also range from 0 to 1. As shown in Table 4, the direct trust between node ‘i’ and node ‘j5’ is the lowest, while the direct trust between node ‘i’ and node ‘j2’ is the highest. This disparity arises from the significant number of failed communications with node ‘j5’ and the substantial number of successful communications with node ‘j2’.

It is worth noticing in Table 3 and Table 4 that the direct trust score among sensor ‘i’ and sensor ‘j1’ remains consistent across both the LTMBE and the BTRES scheme, differing only for the other nodes. This consistency arises because both LTMBE and BTRES employ beta distribution to calculate direct trust scores, and the input data were kept identical to prevent experimental bias. However, the direct trust scores generated by BTRES for nodes j2, j3, j4, and j5 differ from those of LTMBE. This disparity is due to BTRES introducing an additional parameter, $W_{age}$, which is applied to every update of the direct trust score.

After calculating the direct trust scores, we proceed to compute the indirect trust scores, as presented in Table 5. Similar to the LTMBE scheme, indirect trust in BTRES also falls within the range of 0 to 1. It is determined by evaluating the direct interactions between the five recommenders (k1, k2, k3, k4, and k5) and node ‘j1’. These direct evaluations are then weighted by a factor referred to as the recommender weight $\left(\gamma_{k}\right)$.

For both the LTMBE and BTRES schemes, we assumed the presence of only five recommending nodes between node ‘i’ and node ‘j1’. However, in the case of BTRES, the indirect trust is calculated for each trustee if there is at least one recommending node between the trustor and the trustee. This aspect makes the BTRES scheme computationally more demanding compared to LTMBE. In LTMBE, the indirect trust is only computed when the confidence interval value falls below a specific threshold, rendering the scheme lightweight.

Analyzing Table 5, we can observe that the indirect trust of recommender node $k4\;({IT}_{k4j})$ and its associated recommender weight $\left(\gamma_{k}\right)$ are notably lower than those of other indirect trust scores. This discrepancy arises from the fact that recommender node k4 experienced a significant number of failed communications with both nodes ‘i’ and ‘j1’. However, in comparison to LTMBE, BTRES assigns a considerably lower weight to recommender k4. This suggests that BTRES performs more effectively in identifying malicious recommenders that may furnish false data or trust values.

#### 4.3.3. LDTS

In contrast to the LTMBE and BTRES schemes, the LDTS scheme incorporates a WSN topology organized into clusters. These clusters consist of three essential components: a cluster head, its cluster members, and a base station. The LDTS scheme involves two primary forms of communication: intra-cluster and inter-cluster communication. Intra-cluster trust encompasses two distinct types: (1) CM-CM direct trust and (2) CH-CM feedback trust, which can also be considered indirect trust. Similarly, inter-cluster trust comprises two categories: (1) CH-CH direct trust and (2) BS-CH feedback trust, which likewise includes indirect trust.

**Figure 6:**
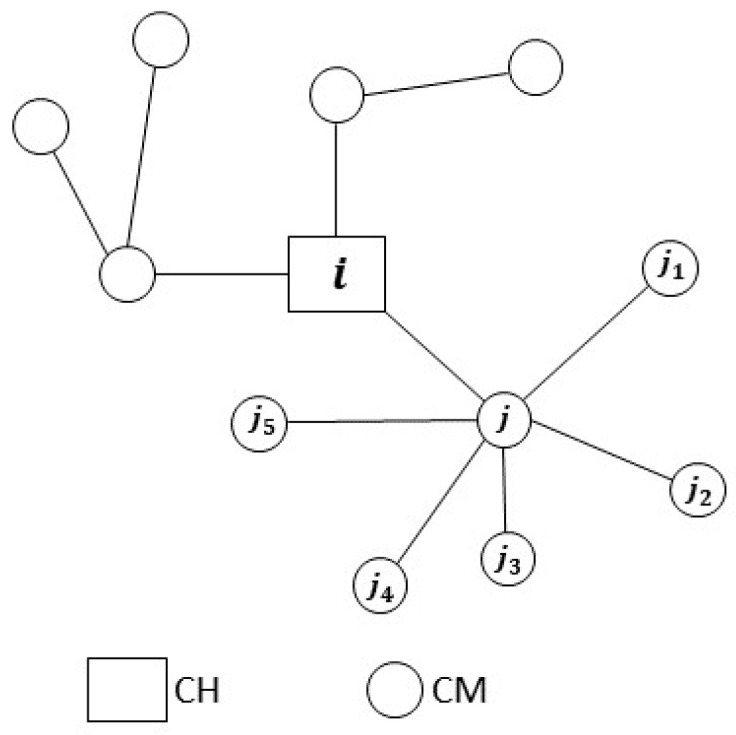
Intra-cluster trust configuration.

**Figure 7:**
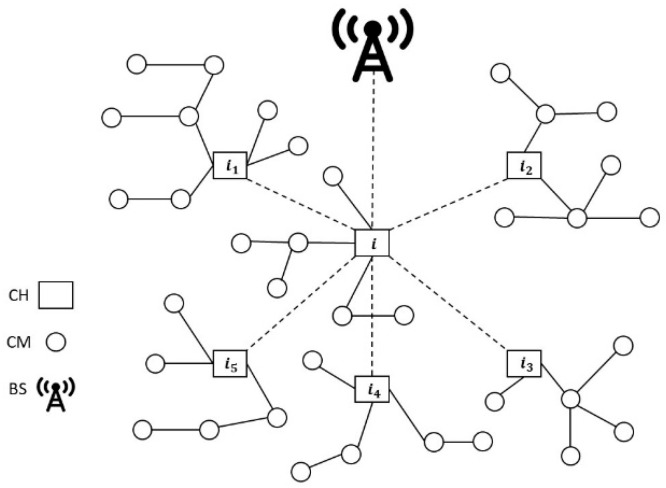
Inter-cluster trust configuration where CH i is directly engaging with CHs i1, i2, i3, i4, and i5.

To implement, analyze, and compare the LDTS scheme with the other two schemes, we introduce specific assumptions. Within the context of intra-cluster trust, we consider a cluster head ‘i’ with six cluster members: j, j1, j2, j3, j4, and j5 where CM j is directly interacting with the CMs j1, j2, j3, j4, and j5 as shown in Figure 6. For the inter-cluster trust, we focus on six CHs: i, i1, i2, i3, i4, and i5, which engage in direct interactions with each other, and the BS as shown in Figure 7. Furthermore, we modify the trust evaluation equations of LDTS by removing the nearest integer function and dividing the results by 10. This adjustment ensures that the trust score range falls between 0 and 1, aligning it with the LTMBE and BTRES schemes. To facilitate a meaningful comparison of direct trust with LTMBE and BTRES, we exclusively consider CM-CM direct trust, omitting CH-CH direct trust since CHs are high-power aggregator devices with substantial storage capacity and computational resources.

1. Intra-cluster Trust

The initial step in the trust evaluation process of the LDTS scheme involves the calculation of CM-CM direct trust, as illustrated in Table 6. Following the computation of direct trust scores, we proceed to determine CH-CM feedback trust. It is noteworthy, as observed in Table 6, that the CM-CM direct trust scores are notably lower in comparison to the direct trust values of the LTMBE and BTRES schemes. Particularly, the CM-CM direct trust value of CM j4 approaches 0. This result can be attributed to the presence of an expression within LDTS direct trust evaluation equation, $\left(1/\sqrt{f_{x,\;y}}\right)$, where $u_{x,}$ represents the number of failed communications. This expression is referred to as the ‘strict punishment feature’ of the LDTS scheme, as it significantly depends on the number of failed communications.

In contrast to LTMBE and BTRES, LDTS does not calculate an individual indirect trust score for each node. Instead, it allows for the calculation of an overall indirect trust referred to as the CH-CH feedback trust score, which is comparable to the overall indirect trust score of LTMBE and BTRES. In terms of energy efficiency, it is important to note that LDTS proves to be the most computationally demanding scheme when compared to LTMBE and BTRES. This heightened computational cost can be attributed to the presence of multiple CHs and the BS, which are high-power devices equipped with substantial storage capacities, unlike sensors.

2. Inter-cluster Trust

In the evaluation of inter-cluster trust, our initial step involves the computation of CH-CH direct trust, as illustrated in Table 7. The calculation process for CH-CH direct trust closely resembles that of CM-CM direct trust, with the distinction being that the communications take place between CHs. Following the computation of CH-CH direct trust scores for the five CHs, we proceed to calculate an indirect trust score known as BS-CH feedback trust.

It is noteworthy that trust scores for inter-cluster trust communication, which includes CH-CH direct trust and BS-CH feedback trust, are significantly lower when compared to intra-cluster trust and the trust evaluations of LTMBE and BTRES. This difference can be attributed to the utilization of communication data from the LTMBE and BTRES schemes. These data predominantly consist of a high number of failed communications in comparison to successful ones. Furthermore, the ‘strict punishment feature’ of LDTS plays a crucial role in rapidly reducing the trust score in response to an increase in failed interactions. This feature effectively mitigates the risk of sudden cyber-attacks.

## 5. Results and Analysis

In this section, we implement, analyze, and compare the LTMBE, BTRES, and LDTS schemes against two major cyber-attacks: (1) an on–off attack and (2) bad-mouthing attack.

### 5.1. On–Off Attack

To illustrate the potential threat of a malicious node exploiting the dynamic nature of trust through time-domain inconsistency, we consider the ‘on–off attack’. In this attack strategy, a malicious node alternates between behaving cooperatively and maliciously to avoid detection while inflicting harm. To facilitate the comparison of the performance evaluation of the LTMBE, BTRES, and LDTS schemes under such an attack scenario, we establish three specific assumptions and create a scenario as follows:

1. Initial trust values: we initialize an initial direct trust value of 0.5 (neutral) for all three trust management models.
2. Malicious node behavior: We assume that a malicious node initially engages in cooperative behavior with its neighboring nodes for the first 50 interactions, aiming to establish a positive reputation and gain trust. Subsequently, the malicious node shifts its behavior to malicious intent for the next 50 interactions, causing damage and potentially undermining trust. Afterward, it resumes cooperative behavior.
3. Consistent communication data: to demonstrate the on–off attack and ensure impartiality, we maintain consistent communication data across all three trust management models, mitigating any potential bias in the results.

**Figure 8:**
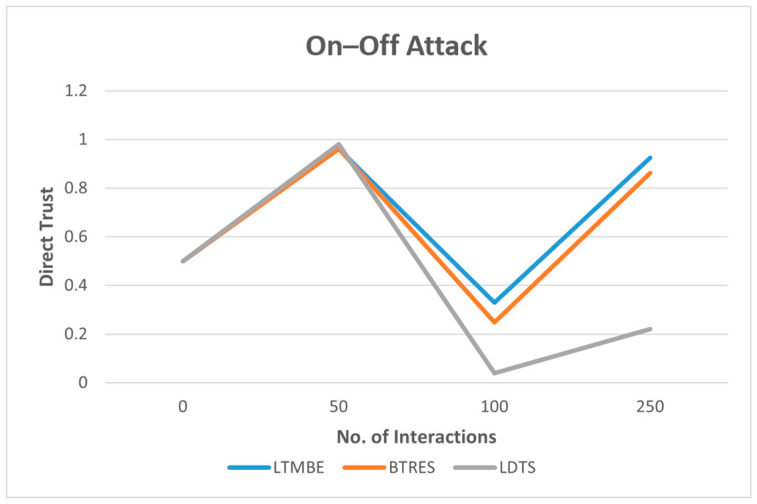
On–Off Attack Results.

The results of the on–off attack are visually presented in Figure 8. It is evident from Figure 8 that in the case of the LTMBE, BTRES, and LDTS schemes, the direct trust at 50 interactions is almost identical and the direct trust of LDTS is the highest at 50 interactions, after which it diverges as the total number of interactions increases. The direct trust score of all three schemes is high at 50 interactions as we assume that all the nodes behave honestly for the first 50 interactions. Moreover, the direct trust score of BTRES and LTMBE is identical at 50 interactions as both the schemes employ the concept of beta distribution to evaluate direct trust. However, the direct trust update processes differ between LTMBE and BTRES.

At 100 interactions, the direct trust score of all three schemes significantly declines as we assume that the sensor nodes behave maliciously from 50 to 100 interactions. Among the three trust evaluation schemes, the direct trust of the LDTS scheme rapidly declines at 100 interactions due to the scheme’s strong sensitivity to the number of failed interactions, influenced by the “strict punishment feature”.

Interestingly, when the total number of interactions reaches 250, the direct trust value rises for all three trust schemes and this behavior aligns with our assumptions. The direct trust significantly rises for the LTMBE and BTRES schemes; however, in the case of the LDTS scheme, the direct trust gradually rises as compared to the other two schemes. This is due to the strict punishment feature of the scheme which severely punishes malicious behavior.

Consequently, we can infer that LDTS demonstrates superior security against the on–off attack compared to the other two trust management schemes. This is attributable to LDTS’s high dependence on the number of failed interactions. Even if there is a small change in the number of failed interactions, it will significantly affect the trust score of the LDTS scheme. Furthermore, the time required for trust accumulation in LDTS significantly exceeds the time needed for trust accumulation for the LTMBE and BTRES schemes, contributing to its robustness against such attacks. Figure 8 illustrates this behavior, where it becomes apparent that after 100 interactions, the direct trust values of BTRES and LTMBE increase rapidly, while the increase in the direct trust value of LDTS is slower when compared to BTRES and LTMBE.

### 5.2. Bad-Mouthing Attack

**Figure 9:**
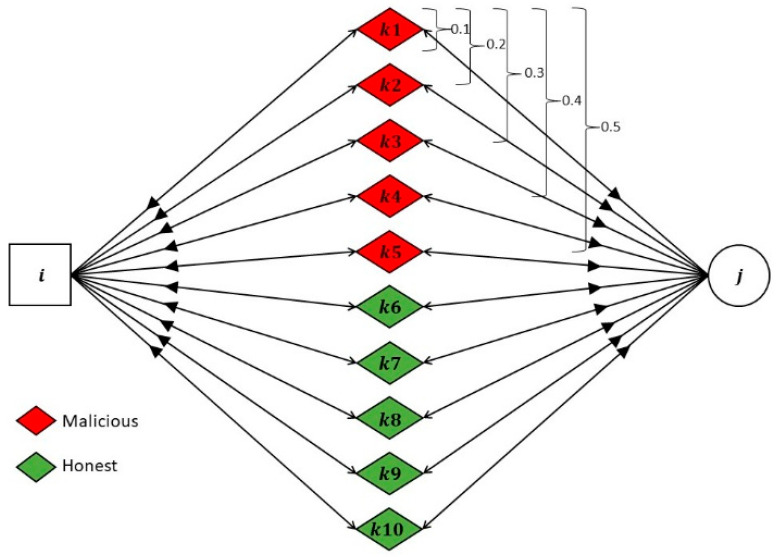
LTMBE and BTRES bad-mouthing attack scenario.

In considering the evaluation of indirect trust, it is imperative to account for the potential threat of a bad-mouthing attack. In a bad-mouthing attack, a malicious node may intentionally provide dishonest recommendations to either frame honest nodes or artificially inflate the trust scores of malicious neighbor nodes. To demonstrate this attack and facilitate a comparative performance assessment of the LTMBE, BTRES, and LDTS schemes, we construct a scenario and establish specific assumptions. For the trust evaluation schemes LTMBE and BTRES, we make the following assumptions:

1. Node i evaluating node j: We assume that sensor ‘i’ is assessing the trust score of sensor ‘j’, and there are ten recommending nodes k1−k10 positioned between them. These ten recommenders deliver feedback about node ‘j’ to node ‘i’ as shown in Figure 9.
2. Proportion of malicious nodes: We introduce a parameter termed the ‘proportion of malicious nodes’, which can take values such as 0.1, 0.2, 0.3, 0.4, and 0.5. When the proportion of malicious nodes is set to 0.1, it signifies that recommender node k1 is malicious and provides false recommendations. For a proportion of 0.2, both recommender nodes k1 and k2 engage in malicious behavior, providing false recommendations, and so forth. Notably, recommender nodes k6 to k10 consistently behave honestly throughout the evaluation.

**Figure 10:**
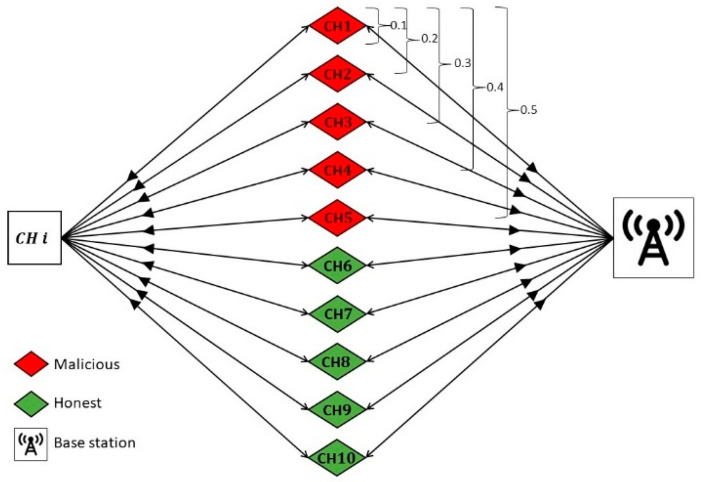
LDTS bad-mouthing attack scenario.

For the trust evaluation scheme LDTS we make the following assumptions:

1. BS evaluating CHs: We assume that CH ‘i’ is evaluating and directly interacting with ten recommending CHs 1–10. However, these 10 recommending CHs and CH ‘i’ are reporting to the BS as shown in Figure 10. In this case, the BS is evaluating the feedback, or the indirect trust, known as BS-CH feedback trust.
2. Proportion of malicious CHs: We introduce a parameter termed the ‘proportion of malicious CHs’, which can take values such as 0.1, 0.2, 0.3, 0.4, and 0.5. When the proportion of malicious CHs is set to 0.1, it signifies that recommender CH 1 is malicious and provides false recommendations. For a proportion of 0.2, both recommender CHs 1 and 2 engage in malicious behavior, providing false recommendations, and so forth. Notably, recommender CHs 6 to 10 consistently behave honestly throughout the evaluation.

These assumptions made for the LDTS scheme are slightly different to the assumptions made for the LTMBE and BTRES schemes. This is due to the cluster-based WSN topology used in the LDTS scheme which differs from the WSN topology employed in the LTMBE and BTRES schemes. However, it is important to note that the logic, the parameters, and the objective in these assumptions are the same for all three trust evaluation schemes.

**Figure 11:**
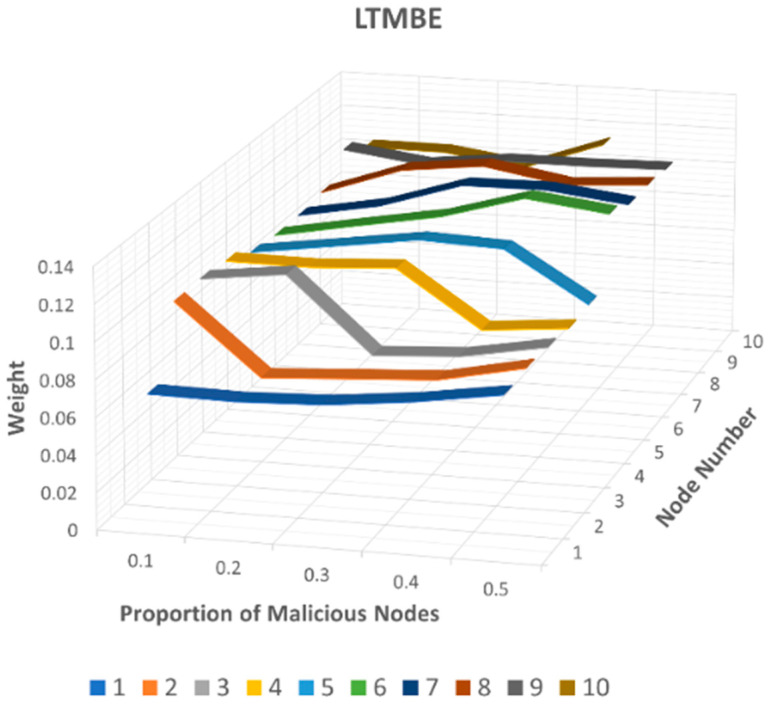
LTMBE bad-mouthing attack results.

**Figure 12:**
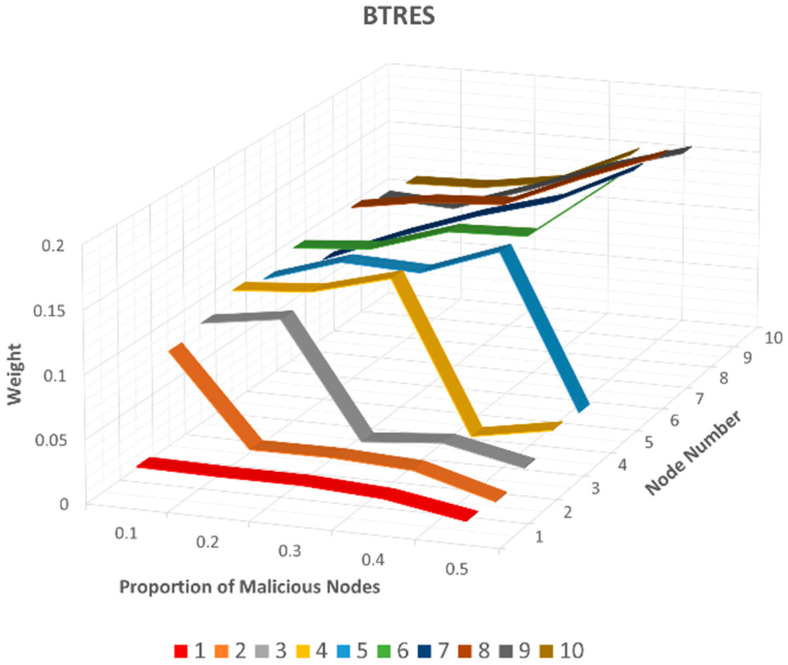
BTRES bad-mouthing attack results.

**Figure 13:**
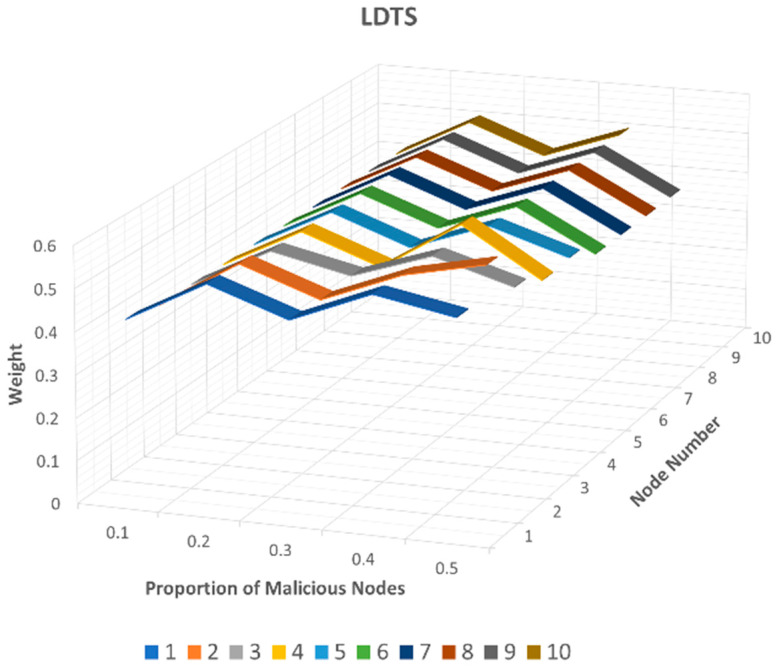
LDTS bad-mouthing attack results.

To assess this attack and compare the weight values assigned to these recommending nodes across all three trust management models, we present the results in Figure 11 (LTMBE), Figure 12 (BTRES), and Figure 13 (LDTS).

Figure 11 illustrates the bad-mouthing attack results for LTMBE, where we can observe that the weight of recommender node 1 consistently remains low for each malicious node proportion value. Additionally, for recommender nodes 2, 3, 4, and 5 in the LTMBE scheme, the weight values experience rapid declines when the malicious node proportion is set at 0.2, 0.3, 0.4, and 0.5, respectively. Furthermore, it is evident from Figure 11 that the weight values of nodes 6 to 10 consistently remain high, aligning with our assumptions. This behavior effectively detects malicious recommenders that provide false recommendations by rapidly diminishing the weight assigned to such deceptive feedback.

Figure 12 reveals that the trends in the results of the bad-mouthing attack for BTRES closely resemble those of the LTMBE scheme. However, an interesting distinction emerges when we consider recommender nodes 2, 3, 4, and 5 in the BTRES scheme, where the weight values experience a rapid drop when the malicious node proportion reaches 0.2, 0.3, 0.4, and 0.5, respectively. This rapid decrease in weight is notably more noticeable than in the LTMBE scheme. Furthermore, it is worth noting that the weight assigned to the honest recommending nodes 6, 7, 8, 9, and 10 in the BTRES scheme consistently exceeds that in the LTMBE scheme.

As a result, we can reasonably infer that BTRES exhibits an effective defense against the bad-mouthing attack and outperforms the LTMBE scheme in this context.

The results displayed in Figure 13 for the LDTS scheme exhibit severe dissimilarities when compared to the LTMBE and BTRES schemes. Notably, the weight values assigned to the honest recommending nodes 6, 7, 8, 9, and 10 are significantly lower in LDTS compared to both LTMBE and BTRES. In some instances, the weight values of these honest recommenders even fall below those of the malicious recommenders 1, 2, 3, 4, and 5, respectively. Additionally, it can be seen from Figure 13 that most of the weight values of the honest recommenders are similar to the malicious recommenders.

Based on this comparative analysis, we can reasonably infer that LDTS cannot effectively defend against a bad-mouthing attack. In contrast, BTRES stands out as the most resilient scheme in countering bad-mouthing attacks when compared to both LTMBE and LDTS.

Based on the empirical evaluation of the three trust evaluation frameworks, we can infer that, for applications requiring energy-saving and efficiency, LTMBE is the most suitable trust management model. This is because the trust evaluation scheme of LTMBE is lightweight, calculating indirect trust only when the direct trust value falls below the confidence level threshold.

However, for WSN applications that demand critical security, BTRES and LDTS emerge as the most suitable trust evaluation models. Among these, BTRES proves to be a superior option compared to LDTS, as it effectively defends against both on–off attacks and bad-mouthing attacks. Additionally, BTRES outperforms the LTMBE model against both on–off attacks and bad-mouthing attacks. Moreover, the BTRES scheme exhibits superior performance compared to the LDTS model when tested against bad-mouthing attacks. On the other hand, LDTS performs the best against on–off attacks among all three trust evaluation schemes. This is attributed to the strict punishment feature of the LDTS scheme, which penalizes malicious behavior and appropriately rewards honest behavior. Table 8 shows the comparison of the three trust management models based on the performance evaluation and their characteristics.

## 6. Conclusions and Future Work

Trust evaluation plays a crucial role in Wireless Sensor Networks (WSNs), ensuring operational reliability and the integrity of sensor nodes by detecting and mitigating malicious entities. However, the absence of comprehensive trust-related datasets and a clear understanding of trust assessment schemes prompted our investigation through a comparative analysis. This research aimed to address these challenges by implementing, comparing, and analyzing three trust evaluation frameworks for WSNs. We thoroughly examined and compared three trust evaluation frameworks for WSNs: LTMBE, BTRES, and LDTS, focusing on their performance in mitigating two major cyber-attacks, namely, on–off attacks and bad-mouthing attacks. Our experimental evaluations revealed insights into their effectiveness and suitability for different application scenarios. Based on our findings, we conclude that the LTMBE trust management model excels in applications prioritizing energy efficiency and conservation. Conversely, for WSN deployments demanding robust security measures, BTRES emerges as the most suitable trust evaluation model. The comparative assessment conducted in this study serves as a benchmark for future research on trust evaluation models in WSNs. Our findings provide valuable guidance for practitioners and researchers alike, informing the selection and development of trust management solutions tailored to specific application requirements. We envision that our work will inspire further exploration and refinement in this critical area of WSN research.

For our future work, we will propose a novel trust evaluation framework and a WSN topology that will leverage sensor semantics and data trust along with communication trust. Unlike the current trust evaluation models that strongly rely on communication trust, our future trust management model will employ data trust to compute the final trust score of sensor nodes in a WSN.

## Figures and Tables

**Table 1:** Communications between sensor I and sensors j1, j2, j3, j4, and j5.

Node	Successful Communications (α)	Failed Communications (β)
j1	4	1
j2	12	2
j3	27	8
j4	9	36
j5	41	10

**Table 2:** Direct trust and confidence intervals of sensor i with 5 other sensors.

Sensor	Direct Trust	Confidence Interval (γ)
j1	0.7143	0.6288
j2	0.8125	0.9709
j3	0.7568	0.9964
j4	0.2128	0.9990
j5	0.7925	0.9995

**Table 3:** Indirect trust values.

Recommender	Indirect Trust	Entropy	Weight	Overall Indirect Trust
k1	0.5635	0.9883	0.2014	0.560
k2	0.6083	0.9659	0.2154	
k3	0.5804	0.9964	0.2065	
k4	0.4412	0.9990	0.1687	
k5	0.5852	0.9995	0.208	

**Table 4:** BTRES direct trust and aging weight values.

Sensor	Direct Trust	$\mathrm{Aging}\;\mathrm{Weight}\;(W_{age})$
j1	0.7143	0.9
j2	0.8129	0.9
j3	0.7528	0.9
j4	0.5447	0.9
j5	0.409	0.9

**Table 5:** Indirect trust and weights.

Recommender	Indirect Trust	Recommender Weight	Overall Indirect Trust
k1	0.1327	0.2172	0.5835
k2	0.1592	0.247	
k3	0.1339	0.2301	
k4	0.0125	0.0647	
k5	0.1471	0.241	

**Table 6:** Intra-cluster trust scores.

CM	CM-CM Direct Trust	CH-CM Indirect Trust
j1	0.8	0.5455
j2	0.6061	
j3	0.2727	
j4	0.0333	
j5	0.2542	

**Table 7:** Inter-cluster trust scores.

CH	CH-CH Direct Trust	BS-CH Indirect Trust
i1	0.1376	0.0726
i2	0.1681	
i3	0.1247	
i4	0.0271	
i5	0.1309	

**Table 8:** Comparison of three trust management models.

Trust Evaluation Schemes	Energy Efficiency	Security against On–Off Attack	Security against Bad-Mouthing Attack
LTMBE	High	Moderate	Moderate
BTRES	Low	Moderate	High
LDTS	Moderate	High	Low

## Data Availability

The data supporting the conclusions of this article will be made available by the corresponding author upon request.
